# The roles of protein expression in synaptic plasticity and memory consolidation

**DOI:** 10.3389/fnmol.2014.00086

**Published:** 2014-11-12

**Authors:** Tali Rosenberg, Shunit Gal-Ben-Ari, Daniela C. Dieterich, Michael R. Kreutz, Noam E. Ziv, Eckart D. Gundelfinger, Kobi Rosenblum

**Affiliations:** ^1^Sagol Department of Neurobiology, University of HaifaHaifa, Israel; ^2^Institute for Pharmacology and Toxicology, Otto-von-Guericke UniversityMagdeburg, Germany; ^3^Research Group Neuralomics, Leibniz Institute for NeurobiologyMagdeburg, Germany; ^4^Center for Behavioral Brain SciencesMagdeburg, Germany; ^5^Research Group Neuroplasticity, Leibniz Institute for NeurobiologyMagdeburg, Germany; ^6^Network Biology Research Laboratories and Faculty of Medicine, Technion – Israel Institute of TechnologyHaifa, Israel; ^7^Department of Neurochemistry and Molecular Biology, Leibniz Institute for NeurobiologyMagdeburg, Germany; ^8^Medical School, Otto von Guericke UniversityMagdeburg, Germany; ^9^Center for Gene Manipulation in the Brain, University of HaifaHaifa, Israel

**Keywords:** protein expression, translation regulation, synaptic stability, synapse, learning, memory consolidation

## Abstract

The amount and availability of proteins are regulated by their synthesis, degradation, and transport. These processes can specifically, locally, and temporally regulate a protein or a population of proteins, thus affecting numerous biological processes in health and disease states. Accordingly, malfunction in the processes of protein turnover and localization underlies different neuronal diseases. However, as early as a century ago, it was recognized that there is a specific need for normal macromolecular synthesis in a specific fragment of the learning process, memory consolidation, which takes place minutes to hours following acquisition. Memory consolidation is the process by which fragile short-term memory is converted into stable long-term memory. It is accepted today that synaptic plasticity is a cellular mechanism of learning and memory processes. Interestingly, similar molecular mechanisms subserve both memory and synaptic plasticity consolidation. In this review, we survey the current view on the connection between memory consolidation processes and proteostasis, i.e., maintaining the protein contents at the neuron and the synapse. In addition, we describe the technical obstacles and possible new methods to determine neuronal proteostasis of synaptic function and better explain the process of memory and synaptic plasticity consolidation.

## INTRODUCTION

Memory can be defined as storage of information manifested as changes over time in the physiology or behavior of an organism in response to environmental stimuli (Crystal and Glanzman, 2013). This definition encompasses not only multi-cellular organisms with evolved brains but also bacteria and plants (Song et al., 2013; Stock and Zhang, 2013). In this review we will focus on organisms in which memory processes are largely mediated by complex nervous systems. In such organisms, memories can be retained for seconds to years, with memory persistence strongly affected by the complexity of the organism’s behavioral repertoire and nervous system, the attention paid to a given experience and the positive or negative value the organism assigns to it by way of interpretation. Indeed, experiences that are of consequence to the organism’s survival, such as assessment of the safeness or toxicity of food, or experiences associated with pain, are learned quickly, and persist for long time periods – in many cases the life span of the organism (Johansen et al., 2011; Gal-Ben-Ari and Rosenblum, 2012).

In organisms with complex nervous systems, memory storage is believed to be heavily based on changes in synapses (Martin et al., 2000; Martin and Morris, 2002), specialized sites of cell–cell contact that connect the nerve cells within the nervous system. Although changes in synaptic connections, broadly referred to as synaptic plasticity, represent only one of multiple neuronal plasticity processes [which include, among others, changes in neuronal excitability, adult neurogenesis, and large scale changes in cortical maps (Giese and Mizuno, 2013)], synaptic plasticity has received the most attention. This attention is justified by the functional potency and enormous flexibility offered by changes at these strategic locations, ideas that can be traced back to the writings of James, Cajal, Freud, and most influentially, Hebb (Berlucchi and Buchtel, 2009; Schott, 2011).

As functionally appealing as these ideas are, nervous systems of all organisms are constrained by their underlying biology, and synapses are no exception. Synapses are composed of proteins, some of which play direct roles in synaptic transmission, whereas others regulate synaptic function or serve as structural scaffolds. Proteins, including synaptic ones, have finite lifetimes and, therefore, need to be continuously replaced with freshly synthesized copies. Moreover, functional changes in particular synapses call for changes in their proteinaceous contents in terms of amounts, distribution, and post-translational modifications. Both synaptic protein homeostasis (proteostasis), as well as functional changes in synaptic protein content present daunting challenges from the perspective of the neuron: the number of synapses is typically huge, their molecular makeup is extraordinarily complex, and their distance from the cell body, where most protein synthesis occurs, can be enormous, in comparison to the average size of a neuronal soma. The regulation of synaptic protein contents is further challenged by the requirement for high fidelity maintenance and trafficking processes essential to minimize spurious changes in synaptic properties, preserve changes induced by physiologically relevant signals, and introduce changes only when and where changes are called for. As a result, the ability of synapses to preserve their individual characteristics for long durations or to modify them in response to physiological stimuli is not obvious at all.

Generally speaking, biological processes mediating memory formation involve numerous tightly regulated molecular and cellular events. These include mRNA transcription; protein synthesis (mRNA translation); mRNA and protein degradation; mRNA and protein trafficking; post-translational modifications such as phosphorylation and ubiquitination; and epigenetic mechanisms, e.g., histone acetylation, DNA methylation, and miRNA regulation (Elkobi et al., 2008; Barki-Harrington et al., 2009; Belelovsky et al., 2009; Gal-Ben-Ari et al., 2012; Gildish et al., 2012; Giese and Mizuno, 2013; Graff and Tsai, 2013; Jarome and Helmstetter, 2013; Saab and Mansuy, 2014). Such processes can be brain hemisphere- and brain subregion-specific (Inberg et al., 2013; Chinnakkaruppan et al., 2014). In following with the term “consolidation” introduced by experimental psychologists (Lechner et al., 1999), the transfer from short-term memory (STM; minutes to hours) to long-term memory (LTM; days to a life time) during which a memory becomes less labile and sensitive to various types of physical or chemical disruption is termed “molecular memory consolidation” (McGaugh, 2000; Kandel, 2001; Dudai, 2004; Merhav and Rosenblum, 2008; Alberini, 2011). The duration of molecular memory consolidation, which varies among different behavioral paradigms and species, is biochemically defined by dependence on unperturbed protein synthesis in the relevant brain regions (Davis and Squire, 1984; Matthies, 1989a,b; Rosenblum et al., 1993; Meiri and Rosenblum, 1998; Costa-Mattioli et al., 2009). In contrast to LTM, STM is not dependent on protein translation (Houpt and Berlin, 1999).

The sensitivity of memory consolidation to manipulations that suppress protein synthesis has important parallels in the most widely studied experimental models of synaptic plasticity, namely long-term potentiation (LTP) and long-term depression (LTD). Synaptic changes induced by LTP and LTD are observable within seconds or minutes of their induction, but their persistence beyond a few hours seems to be strongly dependent on protein synthesis (Nguyen et al., 1994; Rosenblum et al., 2000) but see (Abbas et al., 2009; Villers et al., 2012). Such long-term changes in synaptic strength are usually referred to as “synaptic consolidation” (Clopath, 2012).

The large body of work on molecular and synaptic consolidation has collectively led to the view that long lasting phases of memory formation are dependent on regulated protein expression. Moreover, this body of work has given rise to several notable themes. The first distinguishes between post-translational modifications underlying STM and early phase LTP and a requirement for new protein synthesis for late phase LTP. The second concerns the existence of temporally restricted time windows during which protein synthesis inhibitors are capable of abolishing late phase LTP or the formation of long term memories. The third distinguishes between rapidly occurring processes based on protein synthesis (translation) and slower processes that invoke unique gene expression programs (transcription). The fourth concerns local (mainly dendritic) protein synthesis and processing that is based on organelles, such as polyribosomes located in the vicinity of synapses, and the fifth concerns the existence of specific molecules and pathways involved in synapse to nucleus communication, activated in response to requirements for “plasticity related” proteins at specific synapses. The common denominator of these themes is the view that LTP (and LTD), and by extension, learning processes, invoke fast (sub second) post translational modifications, followed by translation (global and local) and subsequent gene transcription programs (in the soma) with “plasticity proteins” synthesized in the process acting to consolidate changes at specific subsets of synapses.

Whereas this concept is appealing, there are many indications that it is extremely oversimplified. For example, (1) live imaging studies indicate that many synaptic proteins and organelles continuously move in, out, and between synapses (reviewed in Choquet and Triller, 2013; Ziv and Fisher-Lavie, 2014). How do synaptic protein synthesis and degradation rates compare to their exchange and interchange dynamics? How can synapses maintain their specific properties if their molecular constituents are continuously interchanged among nearby synapses? What are the relative contributions of protein redistribution and protein synthesis to synaptic plasticity? (2) To this day, it remains unclear if protein synthesis is required for the provision of “plasticity specific” proteins or simply for the replenishment of depleted components. Which proteins, if any, are synthesized specifically as part of molecular consolidation processes? Where are these synthesized? How long does it take to traffic them to remote synapses? How are they targeted to specific synapses? What prevents their interchange with nearby synapses once they arrive? (3) The conclusion that long lasting phases of memory formation are dependent on regulated protein expression strongly hinges on pharmacological agents that suppress protein synthesis. How do these agents affect ongoing activity in the intact brain (Sharma et al., 2012)? How does this concur with other findings indicating that the requirement for protein synthesis can be relieved by pharmacological agents that inhibit protein degradation (Fonseca et al., 2006a,b); how specific are the activities of these agents? (4) Synapse to nucleus communication is severely challenged by time and space constraints: How can the minute quantities of signaling molecules released from individual synapses overcome the vast distances from remote synapses to the soma?

In the current review, we will try to address some of these questions, using insights gained from our own work and from the work of many others, focusing, where possible, on general principles rather than detailed molecular descriptions and on issues that still remain open. While in recent years tight reciprocal relationships between neurons and astrocytes have been discovered and the now well recognized concept of the “Tripartite synapse” highlights the importance of glia cells for neuronal function and development (Hama et al., 2004; Elmariah et al., 2005), we will mainly focus on the neuronal part of the synapse. However, these general principles of protein homeostasis are likely to be applicable to both neurons and astrocytes.

## METABOLIC HALF LIFETIMES OF KEY SYNAPTIC PROTEINS

As mentioned above, synaptic proteostasis presents daunting challenges from the perspective of the neuron. It thus stands to reason that metabolic turnover rates of synaptic proteins, i.e., their synthesis and degradation rates, would be relatively slow, to minimize the metabolic load of synaptic proteostasis, to allow long transport times along axons and dendrites, and to reduce the likelihood of inaccurate replacement events. In agreement with this reasoning, older studies based on radio-labeling methods indicated that turnover rates of some presynaptic proteins can be remarkably slow, resulting in half-lives (i.e., the time over which one-half of an initial protein quotient is degraded) of many days and even weeks (e.g., Baitinger and Willard, 1987; Petrucci et al., 1991). More recent studies based on synaptically enriched biochemical preparations (synaptosomes) and radioactive amino acids (typically methionine) have reported half-lives for pre- and postsynaptic scaffolding proteins in the range of several hours (e.g., Ehlers, 2003; Yao et al., 2007). In other studies, in which similar methods were used, much longer half-lives were reported for key synaptic proteins such as PSD-95 (El-Husseini et al., 2002) and the AMPA-type glutamate receptor subunit GluA2 (Kjoller and Diemer, 2000). Until recently, however, a systematic analysis of synaptic protein metabolic turnover kinetics was lacking.

**FIGURE 1:**
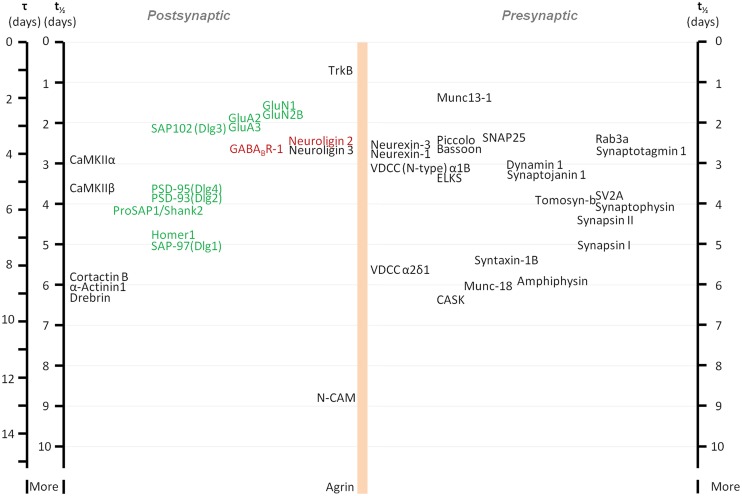
**Metabolic half-life estimates for several well characterized synaptic proteins.** Proteins associated primarily with glutamatergic and GABAergic synapses are shown in green and red, respectively. Note that some related proteins have rather similar half-lives (some proteins with very similar half-lives were separated slightly to increase readability). Adapted from Cohen et al. (2013).

The development of new methods for labeling newly synthesized proteins in cultured cells and in whole animals, combined with mass spectroscopy has allowed an unbiased, systematic measurement of turnover rates of thousands of proteins, including synaptic ones. These studies revealed that turnover rates of synaptic proteins are quite slow, with half-lives on the order of 2 to 5 days in cell culture (Cohen et al., 2013) and probably three to four times longer in adult mice (Price et al., 2010). Some examples of reported half-lives include (in hours, for cell culture/adult mice, respectively) the synaptic vesicle protein Synaptophysin: 98/502; the active zone Bassoon: 62/240; the AMPA type glutamate receptor subunit GluA2: 47/73; the postsynaptic density protein Dlg4 (PSD-95): 88/367; and the protein kinase CaMKIIβ-2: 91/157 (see also **Figure 1**). Interestingly, bioinformatics-based analyses did not reveal significant differences between metabolic turnover rates among presynaptic proteins, postsynaptic proteins, and proteins whose mRNAs are consistently found in dendrites (Cohen et al., 2013). Similarities in turnover were found for some functionally or structurally related proteins (indicative, perhaps of coupled biogenesis, and degradation), but at the same time, proteins belonging to the same compartment could have very different half-lives (Cohen et al., 2013; Toyama et al., 2013). On the whole, the average turnover rate of synaptic proteins in culture was calculated to be on the order of about 0.7% per hour.

While protein synthesis and degradation are undoubtedly essential for synaptic proteostasis, it is important to stress that dynamics of synaptic proteins seem to be dominated by much faster processes, which do not involve the breakdown and synthesis of synaptic molecules, but rather their migration in, out, and between synapses. This conclusion is based on numerous live-imaging studies of multiple synaptic proteins, e.g., neurotransmitter receptors, scaffolding molecules, adhesion molecules, and even synaptic vesicles, which reveal typical residency times of seconds to minutes (receptors, cytoskeletal proteins) to several hours (core active zone and postsynaptic molecules). These studies collectively indicate that synapses are not so much structures in a strict sense, as much as dynamic molecular assemblies at complex steady states (Choquet and Triller, 2013; Ziv and Fisher-Lavie, 2014). This state of things would seem to suggest that the availability of many synaptic proteins might not constitute a limiting factor when rapid changes in synapse composition and size are required, simply because synaptic components (such as vesicles; Darcy et al., 2006) and synaptic molecules could be recruited from nearby synapses (e.g., Krueger et al., 2003; Dobie and Craig, 2011; Mondin et al., 2011). Indeed, a recent detailed analysis of LTP-induced dendritic spine enlargement (Bosch et al., 2014) suggests that this holds true for actin and actin-binding proteins as well as glutamate receptors; on the other hand, the addition of core postsynaptic density proteins (Homer1b, Shank1b) and possibly postsynaptic density enlargement in general do not occur if protein synthesis is blocked, indicating that the *de novo* synthesis of key proteins might be required for synaptic consolidation.

Thus, on the whole, and in line with the *a priori* reasoning described above, the turnover of many synaptic proteins is relatively slow. Yet, it is also important to keep in mind that these estimates were largely based on mass spectroscopy systems, which are inherently biased toward the most prevalent proteins in protein mixtures, and are often blind to cell compartment-specific turnover rates. It thus remains entirely possible that the turnover rates of scarcer synaptic proteins, perhaps proteins that act locally to regulate important synaptic functions, are very different from those described above (see for example Waites et al., 2013). On the other hand, no technique is without shortcomings. Thus, for example, pulse-chase experiments based on radioactive methionine, the canonical method for measuring protein turnover, are typically associated with 100-fold reductions in extracellular concentrations of this essential amino acid. In yeast, similar reductions in extracellular methionine have been recently shown to trigger autophagy (Sutter et al., 2013), raising questions as to the accuracy of turnover rates estimated by this method. Newer methods, such as TimeSTAMP (Butko et al., 2012) are based on measuring degradation rates of fusion proteins (typically expressed under strong promoters), which might differ from those of endogenous forms. At present, therefore, it seems that there is still much uncertainty concerning the metabolic turnover of synaptic proteins and much to learn about how these might be affected by physiological and pathological conditions.

## THE INTERPLAY OF PROTEIN TRANSLATION AND DEGRADATION IN SYNAPTIC PLASTICITY AND CONSOLIDATION

Protein degradation has emerged as one of the mechanisms necessary for memory consolidation (Lopez-Salon et al., 2001; Artinian et al., 2008; Jarome et al., 2011; Reis et al., 2013) and reconsolidation/extinction (Artinian et al., 2008; Lee et al., 2008, 2012), as has been shown using different behavioral paradigms (Jarome and Helmstetter, 2014). The balance between protein synthesis and protein degradation is important for synaptic plasticity, as inhibition of the proteasome or protein synthesis impairs late phase LTP, but co-inhibition of both proteasome activity and protein synthesis has no effect (Fonseca et al., 2006a). Along these lines, it has been shown that LTP increases the rate of protein synthesis, and enhances protein degradation via the ubiquitin-proteasome system (UPS; Fonseca et al., 2006b).

**FIGURE 2:**
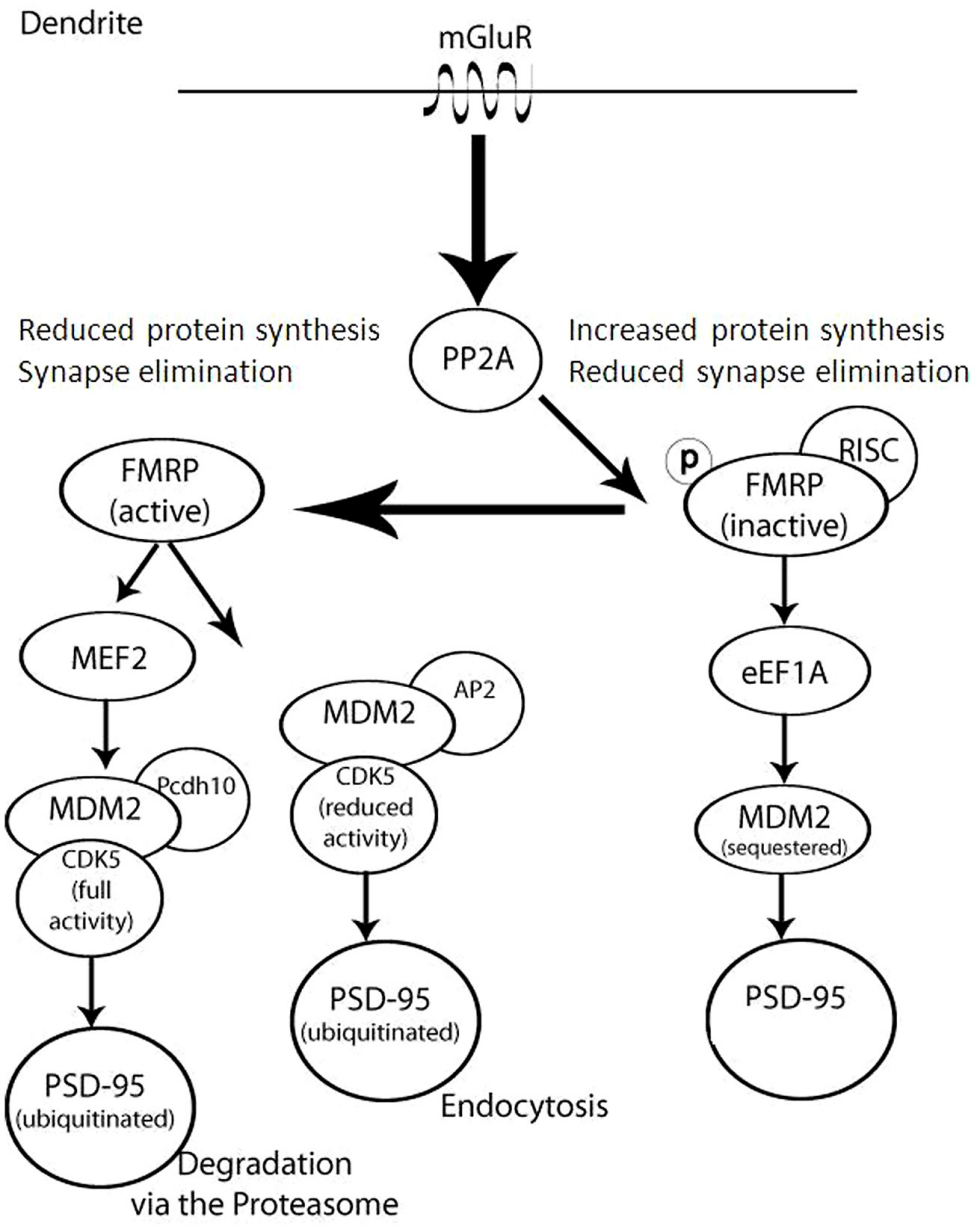
**Complex regulation of FMRP and Dlg4/PSD-95 in dendrites occurs as a result of coordinating translation, degradation, and cellular trafficking.** This model illustrates that in the absence or inactivation of FMRP (right) there is an increase in synaptic protein synthesis and a decrease in synaptic elimination, since PSD-95 is not sequestered or degraded. Upon mGluR activation of PP2A, FMRP dephosphorylation (left) leads to synapse elimination as PSD-95 is either degraded or trafficked out of the synapse.

The significance of both protein synthesis and degradation for synaptic proteostasis is illustrated by fragile X syndrome, manifested by cognitive impairment and increase in dendritic protein translation (Sidorov et al., 2013). At the basis of this syndrome is the loss of fragile X mental retardation protein (FMRP). Phosphorylated FMRP has been shown to bind dendritic polyribosomes, thus stalling protein synthesis (**Figure 2**). FMRP dephosphorylation by activated metabotropic glutamate receptors leads to the dissociation of FMRP from the stalled polyribosomes, thereby increasing rates of protein synthesis. FMRP dephosphorylation also promotes its degradation by the UPS (Nalavadi et al., 2012). Thus, FMRP dephosphorylation both activates translation and promotes the degradation of the protein that suppresses translation, presenting an example of tight coordination between protein translation and degradation.

While the UPS clearly plays essential roles in protein degradation, its involvement in the direct degradation of synaptic proteins is less straightforward. It has been reported several times that treatment with UPS inhibitors can lead to the loss (rather than accumulation) of synaptic proteins (e.g., Ding et al., 2006; Lazarevic et al., 2011; Bajic et al., 2012), possibly by promoting an unfolded protein response (UPR) and the consequential inhibition of protein synthesis (e.g., Ding et al., 2006; Zhang et al., 2010). One notable exception in this regard is the postsynaptic protein GKAP (guanylate kinase-associated protein, also known as SAPAP) which does seem to be directly degraded by the UPS (Ehlers, 2003; Shin et al., 2012). Interestingly, GKAP degradation does not seem to occur *in situ*, as its degradation seems to be preceded by trafficking to the cell body where it is ultimately destroyed.

As mentioned above, memory consolidation is defined as a post-acquisition period sensitive to interference. In many of these studies, this interference was induced experimentally by pharmacological agents that inhibit protein synthesis (Alberini, 2008). Various eukaryotic protein synthesis inhibitors are available, most of which attenuate translation elongation (**Table 1**). However, inhibition of protein synthesis may harbor detrimental effects: in some cases these inhibitors can cause DNA damage, teratogenesis, birth defects, muscle weakness, cardiotoxicity, immunosuppression, and apoptosis. In other cases protein synthesis inhibitors have antitumor effects (Tang et al., 2012). Finally, it has been suggested that these inhibitors can rapidly and acutely suppress spontaneous activity levels (Sharma et al., 2012). For the study of protein degradation, several proteasome inhibitors have been developed (**Table 2**). Here too, however, inhibition of the proteasome may cause cytotoxic effects, lead to apoptosis (Goldberg, 2012), or induce an UPR and a consequential suppression of protein synthesis (Ding et al., 2006; Bajic et al., 2012).

**Table 1:** Protein synthesis inhibitors and their mechanism of action.

Protein synthesis inhibitor	Action
Anisomycin	80s peptidyl transferase inhibitor Tang et al. (2012)
Cycloheximide	Inhibition of eEF2-mediated translocation Alberini (2008)
Diphtheria toxin	Inhibits eEF2 by catalyzing ADP-ribosylation on a diphthamide residue Su et al. (2013)
Emetine dihydrochloride	Inhibition of translocation Alberini (2008)
NSC11989	Preventing the formation of 48s pre-initiation complex Novac et al. (2004)
Puromycin	Amino-acyl tRNA analog, induces premature termination Alberini (2008)
Salubrinal	Inhibits eIF2α phosphatase Wu et al. (2014)
4EGI-1	Inhibition of eIF4E-eIF4G binding and cap-dependent translation initiation Hoeffer et al. (2011)

**Table 2:** Proteasome inhibitors and their mechanism of action.

Proteasome inhibitor	Action
Bortezomib and MG-132	Reversible inhibition of chymotrypsin-like activity of the 20s subunit and caspase-like activity at higher concentrations. Bortezomib is 50 to 100-fold more potent than MG-132 Goldberg (2012)
Lactacystin	Binding to the catalytic core of the 20s subunit, inhibiting all three catalytic activities. Irreversible and fast inhibition of the trypsin and chymotrypsin like catalytic activities Craiu et al. (1997)
Lactacystin clasto-β lactone	The active form of lactacystin Craiu et al. (1997)
Epoxomycin	Binding to the catalytic core of the 20s subunit, inhibiting all three catalytic activities, with higher affinity to the chymotrypsin like catalytic activity Kim and Crews (2013)

Research employing general tools as inhibitors of protein synthesis and the UPS is inherently limited in its ability to enable detailed elucidation of the interaction between protein synthesis and degradation during synaptic plasticity and learning and memory. Therefore, the next level should be studies focused on specific translation regulation factors and their interaction with the degradation machinery. For example, proteasome inhibition-induced enhanced L-LTP is attenuated by interfering with the interaction between eukaryotic initiation factors eIF4E and eIF4G in the hippocampus (Dong et al., 2008, 2014). Blocking of the interaction between eIF4E and eIF4G in the amygdala attenuates long term fear memory consolidation (Hoeffer et al., 2011). Moreover, proteasome inhibition is associated with an increase in expression levels of eIF4E and eukaryotic elongation factor eEF1A (Dong et al., 2008, 2014).

While eEF1A transports aminoacyl tRNAs into the ribosome during translation elongation, its isoform eEF1A2 interacts with newly synthesized proteins (NSPs) and promotes their proteasomal degradation. NSP degradation is increased during stress, following c-Jun *N*-terminal kinase (JNK) phosphorylation of eEF1A2 on Ser205 and Ser358 (Gandin et al., 2013). Translation elongation is also regulated by the phosphorylation of eukaryotic elongation factor 2 (eEF2) and its kinase, eEF2 kinase (Pavitt and Proud, 2009), degraded by the specific E3 ubiquitin ligase SCFβTRCP (Wiseman et al., 2013).

Eukaryotic initiation factor 2α (eIF2α) phosphorylation is increased during aging and neurodegenerative disorders including Alzheimer’s disease and related cognitive impairments (Segev et al., 2013; Ohno, 2014). Heterozygous replacement of eIF2α Ser51 to Ala results in an increase in L-LTP and improved cognitive performance (Costa-Mattioli et al., 2007). One of the kinases regulating eIF2α, protein kinase RNA-like endoplasmic reticulum kinase (PERK), plays an important role in cortical-dependent memory consolidation (Ounallah-Saad et al., 2014). PERK induces an UPR following ER stress, and promotes cell survival via activation of NF-E2-related factor 2 (Nrf2). Nrf2 is a transcription factor activating genes encoding for detoxifying enzymes [e.g., NAD(P)H: quinone oxidoreductase 1 (Nqo1)], but also for the human catalytic proteasome subunit PSMB5 (Cullinan et al., 2003; Lee et al., 2012). Proteasome inhibition may induce prolonged ER stress, and eventually cause apoptosis (Woehlbier and Hetz, 2011), further increased when combined with salubrinal, an inhibitor of the eIF2αs phosphatase, PP1 (Schewe and Aguirre-Ghiso, 2009).

Interestingly, some components of the cellular protein synthesis machinery are homologous to components of the degradation machinery. For example, human eIF3, which participates in the recruitment of the ribosomal 40s subunit and the formation of the pre-initiation complex (Sha et al., 2009), consists of 13 different subunits containing domains which are homologous to the 19s proteasome regulatory particle. Among these is Rpn6, which stabilizes the connection between the 19s regulatory particle and the 20s core particle. Rpn6 shows homology to eIF3 subunits a and c, which participate in the recruitment of the 40s subunit to the ternary complex, but also to subunits e and l (Scheel and Hofmann, 2005; Suo et al., 2011; Pathare et al., 2012). In addition, eIF3e depletion in human mammary epithelium decreases ubiquitin levels as well as functional proteasomes (Suo et al., 2011).

In this part we reviewed some examples how protein translation and UPS-dependent degradation cross-regulate each other. Since tagging with ubiquitin (or ubiquitin-like) moieties can promote various cellular processes in addition to degradation, including cellular signaling as well as protein trafficking, activity, and localization, we assume that an interplay of these processes controls the proteostasis effect on synaptic plasticity and learning processes.

## CHANGES IN THE SYNAPTIC PROTEOME ASSOCIATED WITH PLASTICITY AND MEMORY PROCESSES

The sensitivity of molecular and synaptic consolidation processes to treatments that suppress protein synthesis implies that these processes depend on the availability of NSPs, but their identity remains elusive. It is not even clear if the NSPs are (i) “plasticity-specific” proteins that lead to qualitative changes in the synaptic proteome, or (ii) a manifestation of quantitative changes needed for synaptic growth, or metabolic housekeeping and maintenance, for example.

The search for memory-associated changes in the synaptic proteome is not new, and the results of such searches have been continuously refined by the development of new and more sensitive techniques. Thus, for example, quantitative and qualitative changes in protein expression associated with long-term facilitation in *Aplysia* sensory neurons were studied using two-dimensional gels, resulting in the identification of about 10 individual proteins whose expression levels changed over the course of these experiments (Barzilai et al., 1989). Similarly, significant changes in the protein composition of synaptic junctions following processes of high (pathological) synaptic activity have been reported more than a decade ago (Hu et al., 1998; Wyneken et al., 2001) using micro-sequencing and immunoblots. Since then, mass spectrometry and proteomic methods have been used to characterize the contents of synaptic protein preparations [for review see: (Sheng and Hoogenraad, 2007), in exquisite detail (Chua et al., 2010; Pielot et al., 2012), http://www.synprot.de/], and to follow changes in the proteome in various brain regions in response to memory-relevant plasticity processes. However, most of these studies analyzed changes in whole cell/tissue extracts and, therefore, could only indirectly infer alterations of the synaptic proteome (e.g., McNair et al., 2006, 2007; Monopoli et al., 2011; Hong et al., 2013).

Here, we consider some recent studies that focused on protein fractions enriched for synaptic protein components. An interesting study by Trinidad et al. (2013) reported global activity-dependent changes in the murine synaptic proteome after massive activity onset utilizing the pilocarpine model of epilepsy. They followed the regulation of more than a 100 core protein components of the postsynaptic density that were defined based on previous studies (Sheng and Hoogenraad, 2007; Fernandez et al., 2009) during the first hour after pilocarpine application, assuming that this time window covers mainly the phase of redistribution between synapse-associated and cytoplasmic protein pools. During this initial phase of activity-induced synaptic reorganization, the authors found a relatively tight dynamic co-regulation of a cluster of proteins around α-Amino-3-hydroxy-5-methyl-4-isoxazolepropionic acid (AMPA)- and *N*-methyl-D-aspartate (NMDA)-type glutamate receptors and their scaffolding proteins. Interestingly, isoforms of Ca^2+^/calmodulin-dependent protein kinase type II (CaMKII) displayed a relative down-regulation (10–20% decrease) 60 min after pilocarpin stimulation. The downscaling of this highly abundant protein at the excitatory postsynapse (Kelly et al., 1984) could be indicative of subsequent restructuring processes of synaptic junctions (see also Bosch et al., 2014; Meyer et al., 2014).

**FIGURE 3:**
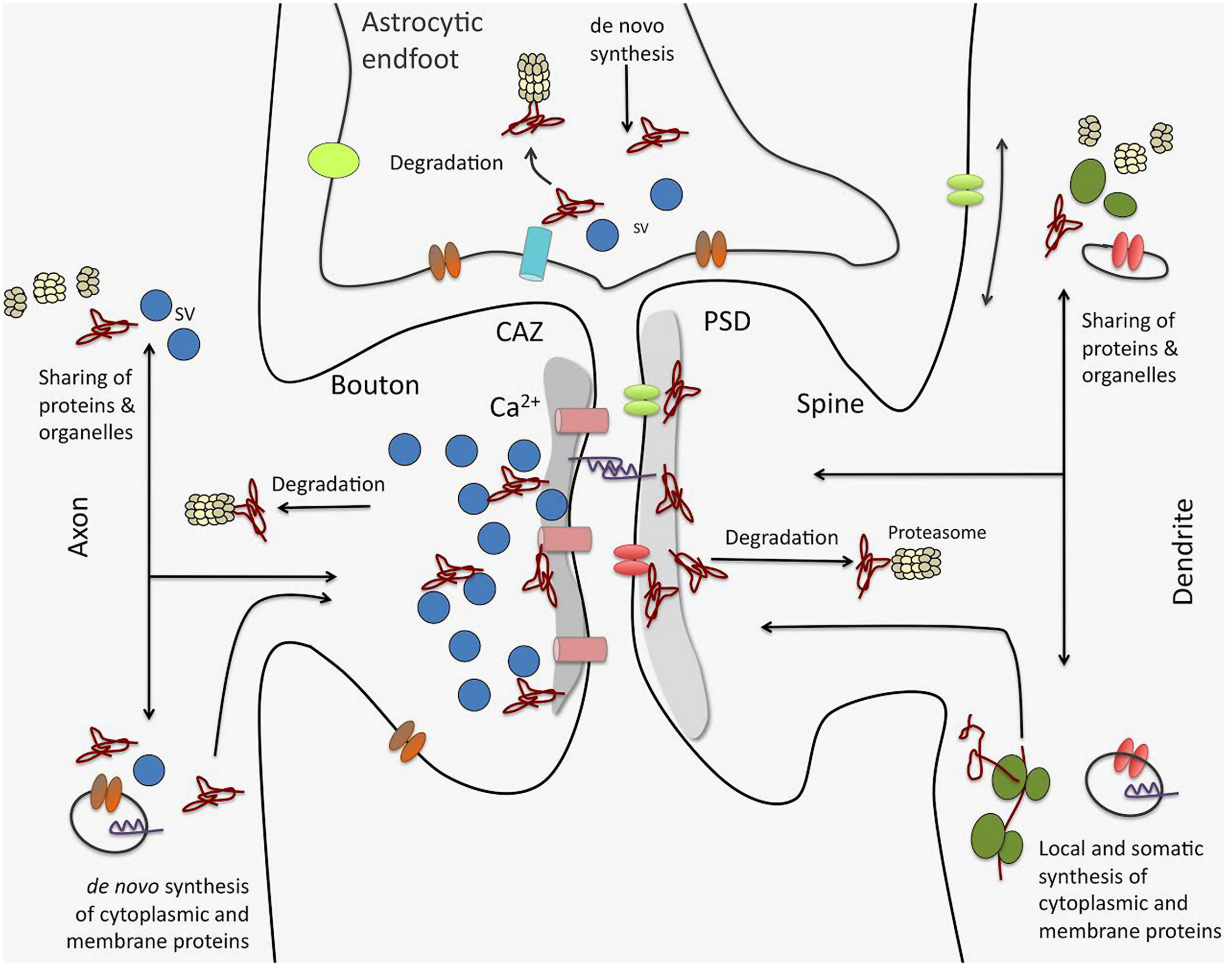
**Processes driving synaptic proteome changes upon memory formation and consolidation.** Mainly three processes acting on both sides of the synapse as well as in astrocytic endfeet intimately contacting synaptic junctions control the turnover of the synaptic protein machinery: (i) *de novo* synthesis of cytoplasmic and membrane proteins that may take place locally or in remote compartments, such as the neuronal soma. This includes both translation by ribosomes as well as complex cellular sorting pathways. (ii) protein degradation, e.g., by the ubiquitin proteasome system, but also by lysosomal and autophagic processes (not shown). And (iii) competitive sharing of proteins, molecular machines (e.g., proteasomes, ribosomes), and organelles between synapses seems to be an important factor in regulating the protein equipment of individual synapses. Sharing of proteins and organelles can occur via intracellular transport processes as well as via diffusion in the cell membrane. Interactive regulation of these processes for protein assemblies and individual proteins is thought to determine protein composition in synapses and thereby govern their individual functionality, and in turn, their contribution to neuronal networks. CAZ, cytomatrix at the active zone; PSD, postsynaptic density; SV, synaptic vesicle.

A recent study on synaptic proteome changes in response to aversively motivated complex auditory learning revealed a surprisingly strong relative down-regulation of synaptic protein components six and 24 h after shuttle box training in four different regions of the mouse brain, i.e., auditory cortex, prefrontal cortex, striatum, and hippocampus (Kahne et al., 2012). Unrelated tone and mild foot-shock stimuli had significantly smaller effects. Down-regulated elements include, among others, large scaffolds on both sides of the synaptic junctions as well as trafficking molecules or components of the UPS. These observations suggest that learning-related plasticity processes as a first step may induce the removal and/or degradation of proteins from both the presynaptic cytomatrix at the active zone and the postsynaptic density, and thereby loosen or ‘deconstruct’ the synaptic structures and prepare them for reconstruction (echoing similar ideas in older studies, e.g., Lynch and Baudry, 1984). Redondo and Morris (Redondo et al., 2010) proposed that this kind of “permissive unlocking” of synaptic structures might be part of the synaptic tagging mechanism (Bosch et al., 2014; Meyer et al., 2014). One of the signaling pathways inducing this unlocking process seems to involve insulin or insulin-like growth factors (Kahne et al., 2012) – factors that have been implicated in a variety of memory processes (Alberini and Chen, 2012). Altogether, these findings suggest that multiple interacting processes (as indicated in **Figure 3**) control the protein levels in the synapse during learning and memory-dependent synaptic plasticity.

Recent developments toward the selective analysis of distinct synaptic subtypes such as the Fluorescence Activated Synaptosome Sorting (FASS) method (Biesemann et al., 2014) or by concentrating on the pool of NSPs using non-canonical amino acids (Dieterich et al., 2006, 2007) may result in a more detailed and focused picture of synaptic proteome alterations during synaptic plasticity events. With bioorthogonal non-canonical amino acid tagging (BONCAT; Hodas et al., 2012) recently were able to identify the dopaminergic subproteome in rat hippocampal neuropil. Interestingly, many of the candidate proteins identified in the dopamine agonist-treated sample belong to gene ontology (GO) categories specific for protein synthesis and synaptic function. In the same study the authors visualized using fluorescent non-canonical amino acid tagging (FUNCAT; Dieterich et al., 2010) an increase in protein synthesis, especially in more distal dendritic regions in dopamine agonist-treated neurons further providing evidence that different cellular compartments may have different needs, in terms of quantity and quality, for NSPs upon synaptic plasticity. By combining both cell-selectivity (such as the FASS methodology) and the selective analysis of NSPs, one might be able to improve our current knowledge of memory-associated changes in the synaptic proteome by identifying even subtle memory-related cell-specific changes in synaptic protein composition. These changes include also alterations in the protein content of, e.g., astrocytic endfeet tightly connected to the presynaptic and postsynaptic sites of synapses (see also **Figure 3**).

## TRAFFICKING OF NEWLY SYNTHESIZED PROTEINS TO SYNAPSES

As mentioned in the introduction, the delivery and removal of proteins to and from synapses is of critical importance for both maintaining and changing synaptic function, yet this necessity is challenged by the huge number of synaptic connections neurons make and receive, the polarized architecture of neurons, the presence of long and branched dendrites and their extraordinarily elongated and extensively bifurcated axonal arbors. Generally speaking, these challenges have been addressed by (1) the development of sophisticated and quite efficient transport mechanisms for delivering particular proteins to the far reaches of the cell and (2) the development of distributed protein synthesis facilities in dendrites and possibly in axons as well (see, e.g., Holt and Schuman, 2013).

While protein synthesis can occur in dendrites and axons (as discussed below), it is generally thought that most synaptic proteins, and in particular presynaptic proteins, are transported from the cell body (e.g., Lee and Hollenbeck, 2003). An intricate transport and processing system mediates proper trafficking and localization of proteins synthesized in the cell body to the synapse and to the extracellular space. During synthesis of proteins in the soma, axonal, and dendritic synaptic proteins are sorted into distinct (dendritic or axonal) vesicles at the *trans*-Golgi membrane owing to distinct sorting peptide motifs, binding to adaptor protein complexes such as AP-4, and other regulatory proteins (Matsuda et al., 2008). Movement of vesicles along microtubule and actin filaments depends on these scaffolding and adaptor proteins that are recognized by molecular motors including kinesins and dyneins (Hirokawa, 1998; Vale, 2003).

Molecular motors involved in transport are sensitive to the polarity of cytoskeletal filaments along which they translocate. Microtubule polarity in dendrites depends on proximity to the cell body: in proximal dendrites microtubule polarity is mixed, whereas in distal dendrites microtubules have a plus-orientation toward the distal tips. In contrast, axonal microtubules are organized uniformly with their plus end toward the distal tips of axons (Baas et al., 1988). Anterograde axonal and dendritic transport of NSPs is mediated by kinesin motors, whereas myosin VI, a minus-end directed motor on actin filaments, is crucially involved in regulating concentrations of axonal proteins (Lewis et al., 2011). Interestingly, vesicles carrying dendritic transmembrane proteins undergo trafficking only within somatodendritic compartments (Burack et al., 2000; Silverman et al., 2001). Recently, the axon initial segment was found to act as a vesicle filter mediating differential trafficking of dendritic and axonal transport vesicles (Al-Bassam et al., 2012). Although vesicles targeted to dendrites entered axons and dendrites with equal proportions, they halted or reversed direction in the axon initial segment in an actin and myosin Va-dependent manner.

The aforementioned, relatively efficient trafficking mechanisms apply mainly to vesicular cargos (as well as mitochondria) and to proteins that (transiently) associate with such cargos. Many other proteins, including synaptic proteins, are trafficked to remote sites by mechanisms collectively referred to as slow axonal transport. As the name implies, trafficking rates can be rather slow, on the order of a few millimeters per day (Hoffman and Lasek, 1975; Vallee and Bloom, 1991; Scott et al., 2011). In fact, when live imaging methods were used to measure the rates at which an axonal protein, e.g., Synapsin (Tsuriel et al., 2006; Tang et al., 2013) or a dendritic protein, e.g., ProSAP2/Shank3 (Tsuriel et al., 2006) were trafficked from the cell body to remote synapses, it was found that their accumulation at remote synapses occurred over many hours and days. These slow trafficking rates pose yet another challenge, as they imply that synaptic proteins must be protected from significant degradation *en route.* Otherwise, sufficient amounts of important synaptic proteins would never reach their remote destinations (a conundrum raised by Alvarez et al., 2000).

The logistic challenges posed by long-range trafficking are partially addressed by the presence of distributed, local facilities for protein synthesis and processing in dendrites and possibly in axons (Holt and Schuman, 2013). In this non-canonical mode of protein synthesis, proteins are synthesized locally by polysomes located in close proximity to synaptic sites. There is increasing evidence that local dendritic protein synthesis allows individual synapses to respond dynamically to the environmental changes that drive the establishment, maintenance, and plasticity of synaptic connections. The same is true for local axonal protein synthesis, which occurs in growing axons to exert axon guidance as well as in mature axons to maintain, for instance, axonal stability and integrity (Jung et al., 2012; Yoon et al., 2012, for a recent review). It is worth noting, however, that while such local protein synthesis systems are usually discussed in the context of synaptic plasticity, perhaps their primary role is to maintain the proteinaceous contents of synapses in face of the logistic challenges associated with long-range protein transport from the remote cell body (Alvarez et al., 2000).

The canonical route by which integral membrane (and secreted) proteins are synthesized, modified, and delivered consists of transport through several membrane-bound structures, including the endoplasmic reticulum (ER), the ER–Golgi intermediate compartment, the Golgi apparatus, the *trans*-Golgi network and vesiculotubular carriers. This raises the question as to how proteins synthesized in remote sites are processed and delivered to the plasma membrane. Golgi outposts have been localized in dendrites (but so far not in axons), concentrating at branch points, where they can participate in post-Golgi trafficking of vesicles (Horton and Ehlers, 2003; Horton et al., 2005), as well as potentially in trafficking and maturation of dendritically synthesized proteins (reviewed in Hanus and Schuman, 2013). But how can selective targeting of NSPs to a subset of modified synapses be achieved with a continuous somatodendritic ER? The ER contributes to various forms of synaptic plasticity through Ca^2+^ release, but also via ER-formed organelles (spine apparatus organelle) composed of stacked smooth ER, which are found in dendritic spines (Spacek and Harris, 1997). The spine apparatus is known to enter and leave, change position and size in a subset of spines (Maggio and Vlachos, 2014). The position and size of the spine apparatus is thought to affect Ca^2+^ homeostasis in the spine compartment, thereby influencing the ability of individual synapses to undergo plasticity. However, further studies are necessary to elucidate the detailed mechanism underlying this effect. Selective targeting of NSPs to a subset of modified synapses is also achieved by microtubule-dependent transport of vesicles. Notably, vesicles carrying different receptors, such as NMDA and AMPA receptors, can be sorted into distinct pools taking different routes to the plasma membrane. While AMPA receptors take the canonical secretory pathway through the somatic ER and Golgi network, NMDAR-carrying vesicles bypass the somatic Golgi and directly merge with Golgi outposts, depending on the adaptor proteins CASK and SAP97 (Jeyifous et al., 2009). Moreover, Cui-Wang et al. (2012) have previously shown an increased ER complexity at branch points and in the proximity of synaptic spines, which could facilitate protein export and processing at secretory hubs in dendrites. Here, AMPARs were shown to rapidly diffuse within the continuous somatodendritic ER, but were restricted in their movement at sites of extensive ER complexity.

It thus seems that in dendrites, the presence of a plethora of mRNAs (Cajigas et al., 2012), polyribosomes, translation factors, regulatory proteins, and Golgi outposts enables the synthesis of a diverse array of different protein classes including receptors, scaffolding, and signaling molecules, thereby bypassing long-distance trafficking of proteins. Many membrane and secreted proteins, however, are highly glycosylated, and indeed, the glycosylation pattern can serve to distinguish mature from immature proteins. Taking into account the low abundance of Golgi outposts in dendrites (Hanus and Ehlers, 2008), NSPs might not undergo all of the processing steps of the canonical secretory pathway, and therefore, could be functionally different from somatically synthesized ones. Thus, faster and more accurate delivery might be at the expense of functional maturity and protein stability.

At present, the relative fractions of somatic or locally synthesized proteins needed for the initial assembly of synapses, their maintenance and their modification remains unknown. We expect that new methods and approaches, such as those mentioned above, will gradually clarify these matters.

## THE IMPACT OF LONG DISTANCE TRANSPORT OF SYNAPTIC PROTEINS TO THE NUCLEUS ON SYNAPTIC FUNCTION AND MEMORY CONSOLIDATION

Several studies have provided compelling evidence that activity-dependent gene transcription plays an important role in preserving changes in synaptic strength as well as in LTM formation (West and Greenberg, 2011). Apart from fast Ca^2+^ signals that elicit immediate early gene expression on a time scale of minutes (Saha and Dudek, 2013), the long-distance transport of proteins from the synapse to the nucleus has attracted considerable interest in recent years (Jordan and Kreutz, 2009; Karpova et al., 2012; Kaushik et al., 2014). This type of signaling is conceptually appealing because it allows for local encoding of signals at the site of origin and decoding in the nucleus. Yet many questions, factual and conceptual remain: How do synapses differentiate between ongoing synaptic activation and specific activity patterns that drive synapse to nucleus communication? What is the nature of such communication molecules? How do the presumably minute quantities of signaling molecules released from a small number of remote synapses overcome the vast distances from and to the soma? How do they retain their integrity and specific properties along the way?

Recent work has indeed shown that distinct signaling events like activation of synaptic vs. extrasynaptic NMDA receptors (Karpova et al., 2013) or activation of different synaptic kinase and phosphatase pathways (Ch’ng et al., 2012) are encoded locally by phosphorylation of messenger molecules that then transit to the nucleus. Other studies uncovered mechanisms which preserve phosphorylation signals during retrograde transport to the nucleus (Karpova et al., 2013). According to this model, synaptic proteins dock at their target sites following nuclear import, and elicit a genomic response that depends on phosphorylation of crucial phospho-sites (Karpova et al., 2013).

A good example for this type of signaling is the synapto-nuclear messenger protein, Jacob, which enters the nucleus following activation of GluN2B-containing NMDAR at synaptic and extrasynaptic sites. The information thought to be encoded and transduced by Jacob pertains to the origin of NMDAR signals to the nucleus: whether it is synaptic or extrasynaptic (Karpova et al., 2013). Synaptic NMDAR activate ERK, and active ERK then binds to Jacob and phosphorylates a crucial serine at position 180. A stable trimeric complex with proteolytically cleaved c-terminal fragments of the neurofilament α-internexin is then formed, which protects serine 180 phosphorylation and active ERK against phosphatase activity during transport. Zhai et al. (2013) have recently shown that following the induction of LTP in just a few spines, nuclear ERK activity increases with a delay of 30 min. This activation is independent of voltage-dependent calcium channels and membrane depolarization, and it is tempting to speculate that Jacob might bind active ERK and bring it to the nucleus. Nuclear trafficking requires binding to neuronal importins at synapses and in dendrites (Dieterich et al., 2008), which in turn provide an adaptor for association to a dynein motor. Jacob transits to the nucleus via an active retrograde transport along microtubules. Following nuclear entry, Jacob docks a NMDAR-derived signalosome, that might arguably differ between synaptic and extrasynaptic receptors with different consequences for gene expression, to nuclear target sites (Karpova et al., 2013).

A limited number of target interactions, the stability of protein complexes, and their presumably prolonged effect on transcriptional regulation may confer specificity and efficiency to messengers like Jacob. Furthermore, following nuclear import from the synapse, nucleo-cyotplasmic shuttling and the nuclear residing time can be tightly regulated by subsequent neuronal activity as recently shown for CRTC-1 (Ch’ng et al., 2012). What are the molecular underpinnings of such a signaling mechanism? It is well established that NMDARs are crucially involved in learning and memory and play a pivotal role in synapse-to-nucleus communication in pyramidal neurons of the hippocampus. The NMDAR complex is a particularly rich source of synapto-nuclear messengers (Karpova et al., 2012) and it is plausible that the assembly of the transport complex starts in close proximity to NMDARs. A splice isoform of the GluN1 subunit of NMDARs harbors a classic nuclear localization signal sequence (NLS) to which importin-α can be docked (Jeffrey et al., 2009). This interaction is modulated by protein kinase C (PKC), which phosphorylates the NLS region of GluN1 in an activity-dependent manner, making importin-α1 available for nuclear trafficking (Jeffrey et al., 2009). Neuronal importins are present in dendrites and synapses from where they can translocate to the nucleus following NMDAR activation (Thompson et al., 2004; Dieterich et al., 2008), and importin-α can associate with a dynein motor for retrograde transport to the nucleus (Hanz et al., 2003).

A yet unresolved issue is how the transport complex is assembled and how NMDAR signals induce dissociation of these messengers from the synapse. Published evidence suggests that different signals can induce the nuclear import of different synapto-nuclear protein messengers. The transcription factor cAMP response element-binding protein 2 (CREB2), for instance, only translocates from synapse to nucleus after the induction of NMDAR-dependent LTD, but not LTP (Lai et al., 2008). In contrast, Jacob only transits to the nucleus following induction of Schaffer collateral NMDAR LTP but not LTD at CA1 synapses (Behnisch et al., 2011).

Interestingly, the nuclear action of many messengers like CRTC1 and Jacob are related to transcriptional co-activation and repression of CREB (Ch’ng et al., 2012; Karpova et al., 2013). This may reflect the central function of CREB in plasticity mechanisms like LTP and its role in the formation of long-term memories. On the other hand, a systematic analysis of gene expression induced by such signaling mechanisms is still pending. At present it is largely unclear how activity-induced gene expression as such feeds back to synaptic function. It will be challenging to identify the cellular mechanisms for protein transport from synapse-to-nucleus and trafficking of NSPs back to synapses. Additionally, the role of these processes in memory formation merits further investigation.

## TECHNOLOGICAL OBSTACLES AND OUTLOOK

Before concluding, we wish to state the obvious, that advances in our understanding of the subject matter have been made possible by the development of novel methods, but have also been hampered by the inherent limitations of these same methods. Thus, for example, FUNCAT allows for the visualization of NSPs but does not resolve the identity of the NSPs. Methods based on metabolic labeling and mass spectroscopy, such as stable isotope labeling with amino acids in cell culture (SILAC) and MS (for a recent review see Hoedt et al., 2014) have provided unprecedented information on the turnover rates of major neuronal proteins, yet they tend to “miss” less abundant proteins, have poor temporal resolution and practically no spatial resolution. Genetic methods, such as TimeSTAMP (Butko et al., 2012) allow for the visualization of newly synthesized copies of particular proteins and for measurements of their degradations rates, but the physiological relevance of these data are sensitive to the degree to which the regulation of expression as well as the degradation rates of these exogenous fusion proteins mimic those of their endogenous counterparts. This is not to say that many of these impediments will not be overcome. Thus, for example, combining BONCAT with SILAC has given rise to QuaNCAT, which has allowed for quantifying changes in the synthesis of >600 proteins in primary T cells following activating stimuli (Howden et al., 2013). New methods for controlling the translation of particular mRNA transcripts (Cao et al., 2013) or driving the degradation of particular proteins (Renicke et al., 2013) by means of light have been described. New technologies with greater sensitivities, resolving power, and improved spatiotemporal resolution are certain to appear and will, in all likelihood, extend our understanding of relationships between protein metabolism, synaptic stability, and plasticity.

## SUMMARY

At present, the literature contains a huge number of studies concerning relationships between protein metabolism, synaptic maintenance and synaptic consolidation on the one hand, and various memory consolidation processes on the other. The interpretation of the latter in the context of the former has often been confounded by a lack of basic information on key issues, such as the lifetime of neuronal proteins, the dynamics of protein trafficking, the nature of synapse to nucleus communication, the source, repertoire, and amounts of NSPs, their modulation by behavioral, physiological (and non-physiological) manipulations, the actual roles and targets of protein degradation systems, and the full scope of reactions invoked by pharmacological manipulations of protein metabolism. Over the last years, with the development of new techniques and approaches, significant progress has been made. Nevertheless, much is yet to be learned on these and other basic issues if we hope to understand the principles that tie together synaptic protein metabolism, synaptic biology, memory formation, and memory consolidation.

## AUTHOR CONTRIBUTIONS

Tali Rosenberg, Shunit Gal-Ben-Ari, Daniela C. Dieterich, Michael R. Kreutz, Noam E. Ziv, Eckart D. Gundelfinger, and Kobi Rosenblum have contributed equally to this work.

## Conflict of Interest Statement

The authors declare that the research was conducted in the absence of any commercial or financial relationships that could be construed as a potential conflict of interest.
